# The Earthy Phosphates

**Published:** 1884-10

**Authors:** 


### THE EARTHY PHOSPHATES.

It has been, and still is, the custom of many intelligent dentists to prescribe for pregnant women phosphate of lime and other like preparations, in the expectation that they will exercise a beneficial influence upon the teeth of the fœtus. Mothers whose previous offspring had bad teeth were persuaded that the trouble arose from a lack of lime salts, and that all that was necessary was to artificially supply them; indeed we have frequently heard dentists expatiate upon the wonderful results that have sometimes resulted from such treatment. We cannot but think that these cases are only coincidental, and in but few instances due to a course of treatment that seems to us a violation of all physiological law. It is true that in rachitis, for instance, there is a lack of certain known elements, but it is also true that they cannot be mechanically supplied. There has been a great outcry against the use of finely bolted flour, but it has been more than once demonstrated that even were white flour the exclusive diet of a pregnant woman, it contains earthy phosphates far in excess of the actual needs of both mother and fœtus. No matter what the food, almost invariably these are found in the excretions during pregnancy, showing that if there be a lack of such elements in the bones or teeth of the child, the fault lies in the nutritive process; in assimilation, rather than in an imperfect diet. Of course we do not mean to imply that care in the selection of good and wholesome food is a matter of no consequence, for there are other considerations besides those of the mere presence or absence of certain essential ingredients in great or small quantities.

The food of man is originally derived from inorganic materials, but his organization is unable to elaborate it directly. It is the function of vegetable life to organize the inorganic. Plants can obtain nutriment directly from the earth by the assimilation of inorganic matter. Animals exist only by the digestion of organized bodies. The carnivora must even have this organic matter *re*-organized before it will serve for their pabulum, and hence their food must have passed through two processes of digestion. First, it must have been organized by the vegetable kingdom, and this again must be redigested in an animal organization before it is fitted for their nutrition. Man has a digestive apparatus that will accept of pabulum that has been either once or twice organized, but he cannot assimilate inorganic matter.

This being the case, it follows that it is idle to administer earthy matter in the expectation that it will be directly incorporated into the system. The digestive function must elaborate its own material from that which has been once organized before its introduction into the alimentary canal, and therefore the giving of earthy phosphates for the growth of the teeth or bones is a mistaken practice. But we may prescribe inorganic substances with good results where we desire to exercise a definite influence upon certain of the tissues or organs. By their mere presence they may modify or change function, and control or direct imperfect digestion and assimilation, so that the system shall appropriate instead of reject the needed elements of the pabulum. It is possible that phosphate of lime, for instance, may thus act remedially, although it has never been regarded as a medicinal agent of much value. If it be prescribed, therefore, it should be done intelligently, in the expectation of alterative effects, and not ignorantly, with the view of its being directly organized into the osseous system.

